# Renin-Angiotensin System Induced Secondary Hypertension: The Alteration of Kidney Function and Structure

**DOI:** 10.1155/2021/5599754

**Published:** 2021-10-04

**Authors:** Zahra Pezeshki, Mehdi Nematbakhsh

**Affiliations:** ^1^Water and Electrolytes Research Center, Isfahan University of Medical Sciences, Isfahan, Iran; ^2^Department of Physiology, Isfahan University of Medical Sciences, Isfahan, Iran; ^3^Isfahan^MN^ Institute of Basic and Applied Sciences Research, Isfahan, Iran

## Abstract

Long-term hypertension is known as a major risk factor for cardiovascular and chronic kidney disease (CKD). The Renin-angiotensin system (RAS) plays a key role in hypertension pathogenesis. Angiotensin II (Ang II) enhancement in Ang II-dependent hypertension leads to progressive CKD and kidney fibrosis. In the two-kidney one-clip model (2K1C), more renin is synthesized in the principal cells of the collecting duct than juxtaglomerular cells (JGCs). An increase of renal Ang I and Ang II levels and a decrease of renal cortical and medullary Ang 1–7 occur in both kidneys of the 2K1C hypertensive rat model. In addition, the activity of the angiotensin-converting enzyme (ACE) increases, while ACE2's activity decreases in the medullary region of both kidneys in the 2K1C hypertensive model. Also, the renal prolyl carboxypeptidase (PrCP) expression and its activity reduce in the clipped kidneys. The imbalance in the production of renal ACE, ACE2, and PrCP expression causes the progression of renal injury. Intrarenal angiotensinogen (AGT) expression and urine AGT (uAGT) excretion rates in the unclipped kidney are greater than the clipped kidney in the 2K1C hypertensive rat model. The enhancement of Ang II in the clipped kidney is related to renin secretion, while the elevation of intrarenal Ang II in the unclipped kidney is related to stimulation of AGT mRNA and protein in proximal tubule cells by a direct effect of systemic Ang II level. Ang II-dependent hypertension enhances macrophages and T-cell infiltration into the kidney which increases cytokines, and AGT synthesis in proximal tubules is stimulated via cytokines. Accumulation of inflammatory cells in the kidney aggravates hypertension and renal damage. Moreover, Ang II-dependent hypertension alters renal Ang II type 1 & 2 receptors (AT_1_R & AT_2_R) and Mas receptor (MasR) expression, and the renal interstitial fluid bradykinin, nitric oxide, and cGMP response to AT_1_R, AT_2_R, or BK B_2_-receptor antagonists. Based on a variety of sources including PubMed, Google Scholar, Scopus, and Science-Direct, in the current review, we will discuss the role of RAS-induced secondary hypertension on the alteration of renal function.

## 1. Introduction

Hypertension is a chronic medical condition known as a major risk for cardiovascular and chronic kidney disease (CKD) [1]. Primary (essential) hypertension does not have an identifiable reason, and 90 to 95% of hypertensive people have essential hypertension, whereas secondary hypertension develops because of an underlying medical condition or disease and accounts for around 5–10% of the cases [2, 3]. In some conditions, kidneys, heart, or arteries and endocrine systems can induce secondary hypertension. Paying attention to secondary hypertension and choosing the appropriate treatment, secondary hypertension will be controlled, and the underlying diseases and the risk of serious complications such as heart disease, kidney failure, and stroke will be limited [2, 3]. The capability of the renin-angiotensin system (RAS) enzymes or receptor antagonists for the treatment of hypertension reveals the important role of RAS. Physiologically, RAS plays a key role in regulating blood pressure (BP) and electrolytes homeostasis while its activation contributes to the pathogenesis of hypertension [4–6]. RAS-dependent hypertension leads to kidney fibrosis and progressive CKD [4, 5], while the latter is the most common cause of secondary hypertension, and it is an independent risk factor for cardiovascular outcomes [5, 7].

The most important RAS component is angiotensin II (Ang II) which acts mostly via two major receptors of type 1 (AT_1_R) and type 2 (AT_2_R) [8]. AT_1_R and AT_2_R have counterregulatory actions in the cardiovascular and renal systems. The effect of Ang II is mediated primarily by AT_1_R which is expressed in all the cell types within the kidneys [8, 9]. Renal AT_1_R stimulation is essential for the development of Ang II-dependent hypertension [10]. Systemic or renal chronic elevation of Ang II levels via AT_1_R stimulates cellular oxidative injury, reactive oxygen species (ROS) production, apoptosis, vascular inflammation, endothelial dysfunction, chronic heart failure (CHF), and CKD [8, 11, 12]. On the contrary, the AT_2_R opposes the AT_1_R, and its expression has a lower degree than AT_1_R. The physiological action of AT_2_R is not fully known, but it is known to provide a protective effect against hypertension via augmentation of renal bradykinin (BK), nitric oxide (NO), and cGMP production [13–15]. Ang1-7 is also another key component of the RAS that induces vasodilation via its specific receptor named Mas receptor (MasR). It is documented that AT2R and MasR agonists stimulate NO production in proximal tubules [16]. Ang1-7 has counterregulatory role by opposing AT_1_R stimulated vasoconstriction and proliferation, and it affects renal functions and hypertension regulation [17, 18]. Ang1-7 also alters renal hemodynamic responses through increasing renal blood flow (RBF) and decreasing renal vascular resistance (RVR) [19]. An increase of Ang II may affect local renal RAS, and due to counterregulatory effect of hypertension and renal injury, it is necessary to understand the exact renal RAS activity-induced hypertension and its effect on renal functions for possible potent therapeutic targets of hypertension therapy.

In this review, we investigate the clinical and experimental models of Ang II-induced hypertension as secondary hypertension and the factors affected by Ang II-dependent hypertension. The subjects that underling this article are discussed hereinafter in this paper.

- Clinical and experimental models in Ang II-induced hypertension
- Intrarenal renin expression in Ang II-induced hypertension
- Renal and systemic RAS arms modifications in Ang II-induced hypertension
- Intrarenal AGT expression and urinary AGT excretion in Ang II-induced hypertension
- RAS receptors expressions alter in Ang II-induced hypertension
- Inflammation in Ang II-induced hypertension
- Kidney injury markers in Ang II-induced hypertension and the role of fibrotic and inflammatory factors
- Renal interstitial fluid BK, NO, and cGMP responses in Ang II-induced hypertension
- Renal function and hemodynamic response in Ang II-induced hypertension

## 2. Clinical and Experimental Models in Ang II-Induced Hypertension

Various forms of experimental and clinical hypertension can be induced by increasing renin formation in the kidney such as renal artery stenosis induced by atherosclerotic plaques, fibromuscular dysplasia, congenital bands, extrinsic compression, vasculitis, and neurofibromatosis [20]. Clinically, a juxtaglomerular cell (JGC) tumor may induce secondary hypertension via renin production [21]. Experimentally, Goldblatt and Grollman's methods cause Ang II increasing which stimulates the intrarenal and intratubular RAS [22–24]. Three types of hypertension could be performed by Goldblatt methods, including two-kidney one-clip (2K1C), one-kidney one-clip (1K1C), and two-kidney two-clip (2K2C) hypertensions [23–25]. These models of hypertension are performed by constriction of unilateral renal arterial which initiates RAS activation due to an increase in renin secretion [23–25]. In the Grollman model which is also called 2-kidney, 1-shape-of-8-wrap (2K1W), the kidney tissue is wrapped by a shape of 8 ligatures around the kidney and hypertension induced via external compression of renal parenchyma by renin production [14, 24].

## 3. Intrarenal Renin Expression in Ang II-Induced Hypertension

**Figure 1:**
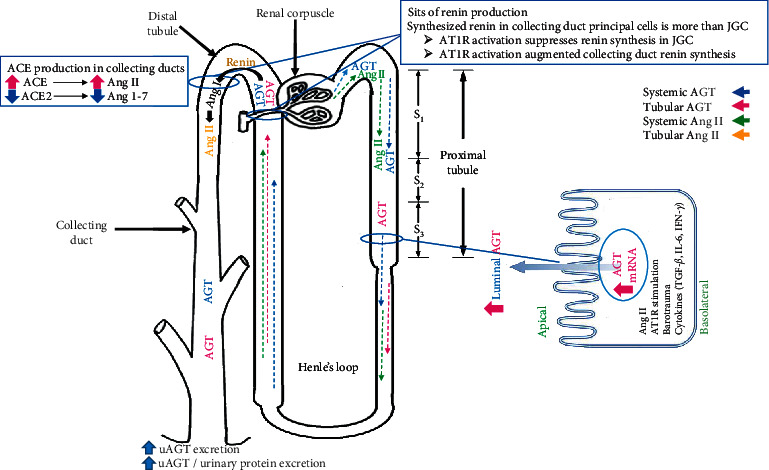
AGT, renin, and ACE production in the renal system in Ang II-dependent hypertension model.

Renin regulates Ang I generation, and JGCs are the primary source of circulating and intrarenal renin production [26, 27]. The plasma level of renin activity increases immediately after initiation of 2K1C and returns to normal after 28 days [25]. Chronic administration of Ang II increases renin mRNA and protein levels of principal cells in the connecting tubules and collecting ducts [28]. The collecting duct renin enhances in both clipped and unclipped kidneys of Goldblatt hypertensive rats [29]. The upregulation of renin expression in both kidneys in the 2K1C model does not depend on blood pressure level [29]. In addition, the synthesized renin in the collecting duct principal cells is more than JGC with different regulation mechanisms [28, 30] (Figure 1). AT1R and renin mRNAs are coexpressed in JGC while AT_1_R alters stimulation of renin synthesis [29, 31]. Ang II acts directly on renal JGC via AT1R and its activation suppresses renin synthesis in JGC [31]. However, Ang II itself can augment the collecting duct renin synthesis [32] via AT1R activation directly (the collecting duct renin synthesis is inhibited by AT_1_R antagonist) [22, 33] (Figure 1).

The collecting duct is also the major source of prorenin in diabetes [32]. The prorenin receptor in the kidneys is localized in the mesangial cells and distal nephron segments, but in the distal segment, it is localized in the intercalated type A cells and not in principal cells [34, 35]. The prorenin receptor gene expression is upregulated in the clipped kidney of the 2K1C model [36] possibly due to the existence of simultaneous upregulation of renin and prorenin receptors in distal segments of the clipped kidney.

Renin and prorenin secretions into the tubular lumen and binding to their receptors enhance the catalytic actions of these enzymes and increase the formation of local Ang I from AGT in the collecting duct [30, 37]. In addition, the increased luminal ACE in the collecting ducts converts Ang I to Ang II [38] (Figure 1). Thus, enhancement of renin synthesized in the kidney under any circumstance contributes to Ang II-dependent hypertension.

## 4. Renal and Systemic RAS Arms Modifications in Ang II-Induced Hypertension

In 2K1C hypertensive models, unilateral renal arterial clipping increases renin secretion; this leads to an increase in circulating renin and Ang II during the early stages. After two weeks, the enhancement of systemic Ang II inhibits renin production in the JGC of the unclipped kidney [23, 25, 39]. The induced 2K1C hypertension elevates the intrarenal Ang II levels in both kidneys [23, 40]. Ang II induces renal and peripheral vasoconstriction which leads to inappropriate activation of the intrarenal/intratubular RAS and progression of kidney disease [22, 41]. The plasma renin activity also increases in the Grollman hypertensive rat model, and it increases Ang II levels in wrapped kidneys [14]. The increased Ang II level in the clipped kidney reveals an increase in renin secretion; however, it is reported that when one kidney is clipped, the AGT mRNA in the contralateral kidney is stimulated, so the possible elevation of intrarenal Ang II in the unclipped kidney is related to stimulation of AGT mRNA in proximal tubule cells [13, 23, 42, 43]. The activation of local luminal AT_1_R in the proximal and distal tubules induces local Ang II formation [41, 42]. The intrarenal RAS and intratubular RAS provoke renal vasoconstriction and enhance tubular sodium reabsorption to maintain sodium balance and blood pressure, while inappropriate RAS activation promotes the development of hypertension and renal damage [22, 23, 44].

During Ang II-dependent hypertension, the upregulation of collecting duct renin production contributes to Ang II/ACE and Ang 1–7/ACE2 balance modification, and this phenomenon reduces the Ang1-7 generation which leads to an increase in intrarenal Ang II [33, 40] (Figure 1). The renal medullary of Ang I and Ang II levels increases, and the renal medullary of Ang 1–7 levels decreases in both kidneys in the 2K1C rat model, and Ang II enhancement is greater in the clipped kidney than in the unclipped one [40]. In addition, the renal cortical Ang I and Ang II levels increase in the clipped kidney but not the unclipped one; moreover, the cortical Ang1-7 level reduces in both kidneys of the 2K1C rat model [40]. An increase of ACE as well as a decrease of ACE2 activities in medullary region of both kidneys and in cortical region of clipped kidney in 2K1C model alter the Ang II level [40] (Figure 1). In the spontaneously hypertensive rat (SHR) model, both cardiac and renal ACE2 mRNA decline [45].

It also is documented that ACE2, prolyl carboxypeptidase (PrCP), and prolyl endopeptidase (PEP) remove the carboxyl-terminal phenylalanine residue to form Ang1–7 from Ang II [46–48], and in physiological conditions, the PrCP is highly expressed in the kidney. In the 2K1C hypertension model, the renal PrCP expression and its activity reduce in the clipped kidney [49], but no alteration in PEP occurs, and the levels of ACE and renin elevate in unclipped and clipped kidneys, respectively [49]. Downregulation of PrCP may attenuate the renoprotective effects of Ang1–7 through reduction of Ang II degradation and cause the progression of renal injury and aggravate damage in the kidney [49]. Both the increase of ACE and decrease of ACE2 levels during Ang II-dependent hypertension lead to the enhancement of intrarenal Ang II and decrease Ang 1–7 contents by reducing the ACE2-mediated degradation of Ang II which leads to the reduction of the protective effects of Ang 1–7 and aggravation in pathological effects of Ang II via AT_1_R activation.

## 5. Intrarenal AGT Expression and Urinary AGT Excretion in Ang II-Induced Hypertension

AGT is expressed in many tissues, including liver, adipose tissue, heart, vessels wall, brain, and kidney, while intrarenal AGT mRNA and protein are localized in proximal tubule cells [13, 50]. AGT protein is found in proximal tubule cells and the cells which are closer to Bowman's space (S1 and S2 segment of proximal tubule) while AGT mRNA is expressed in the S3 segment of proximal tubule cells, and the proximal tubule's AGT within the S1 and S2 segments may originate from the liver, whereas AGT itself synthesizes in the S3 segment [51] (Figure 1). The Ang II enhancement in Ang II-induced hypertension leads to the stimulation of AGT expression and its intrarenal production which increases the urinary AGT (uAGT) excretion [23, 44] (Figure 1). The uAGT excretion rate is a specific index of intrarenal RAS status, and in hypertensive patients, it is greater than normal subjects [44, 52]. In addition, it is reported that the renal AGT mRNA, uAGT excretion rates, and uAGT/urinary protein excretion ratio in unclipped kidneys were greater than clipped kidney in the 2K1C rat model [23].

It is also indicated that intrarenal AGT mRNA level was elevated in the unclipped kidney by 2.15-fold compared to clipped one [53]. As mentioned before, the enhancement of renin secretion increased Ang II level in the clipped kidney, while the elevation of intrarenal Ang II in the unclipped kidney is related to stimulation of AGT mRNA and protein in proximal tubule cells by a direct effect of systemic Ang II level [13, 23, 42, 43]. Ang II enhances AGT mRNA stability and exerts positive feedback on the AGT protein production [54, 55] (Figure 1). The AGT synthesis in proximal tubules is also stimulated by cytokines, including interleukin (IL)-6 [44, 56]. The IL-6 also plays a crucial role in Ang II-induced AGT increase in proximal tubular cells [53, 57] (Figure 1).

The AGT expression increases in glomerular diseases, and AT_1_R antagonist prevents the increase of intrarenal AGT level and consequently reduces the progression of hypertension [58, 59]. The local AT_1_R mediates Ang II uptake by the multiligand endocytic receptor of megalin and caveolin 1-dependent mechanisms in the proximal tubule [60–62]. AT1R stimulation also induces renal cortical mRNA and protein expression of AGT and causes Ang II increment in the proximal tubule [63] (Figure 1). The ACE inhibitors prevent the development of hypertension and proteinuria [6], but AGT may involve both Ang II-dependent and Ang II-independent functions such as body weight gain and liver stenosis [50, 64] but overexpression of AGT in the proximal tubule causes hypertension [65]. One concern about ACE inhibitor treatment is related to the reduction of Ang II level, but the level of AGT is still high and AGT has a greater affinity for renin that may be restored in RAS activity [65, 66]. The transgenic mice with overexpression of specific AGT in the proximal tubule performed a higher blood pressure despite having normal plasma AGT levels and plasma renin activity [65]. Thus, AGT overexpression in the proximal tubule with physiological renin levels (without renin overexpression) results in hypertension and enhanced generation of reactive oxygen species (ROS), NADPH oxidase activity, tubular apoptosis, and tubulointerstitial fibrosis [6, 67], and the elevation of intrarenal AGT protein and AGT mRNA expression affect renal function and degree of injury [23]. To summarize, Ang II-dependent hypertension stimulates AGT production in the S3 segment of the proximal tubule, renin production in collecting duct principal cells, and ACE production in collecting ducts. Ang II-dependent hypertension decreases ACE2 levels leading to Ang II/ACE and Ang 1–7/ACE2 balance modification. AGT production is affected by Ang II, AT1R stimulation, barotrauma, and cytokines (TGF-*β*, IL-6, IFN-*γ*). AGT excretion enhances in Ang II-dependent hypertension (Figure 1).

## 6. RAS Receptor Expressions Alter Ang II-Induced Hypertension

The effects of Ang II and Ang 1–7 are exerted by their specific receptors. AT_1_R, AT_2_R, and MasR are broadly distributed in various regions of the kidney [16, 68, 69]. AT_1_R has the most important action in Ang II-dependent hypertension by inducing intensive vasoconstriction [70]. AT_1_R is localized in renal vascular smooth muscle cells, including afferent and efferent arterioles, vasa recta, mesangial cells, brush border and basolateral membranes of proximal tubule, thick ascending limb epithelia, distal tubule and collecting duct cells, glomerular podocytes, and macula densa cells [71–73]. This receptor has a higher contribution to the vasculature of the renal cortex and the proximal tubules of the outer medulla [9].

The AT_2_R proteins also contribute throughout the rat kidney except in the glomerulus and medullary thick ascending limbs of Henle, and its expression varied throughout life. It is expressed in the fetal and newborn rat kidney and declines in adults; however, AT_2_R is expressed in adults in some conditions such as sodium depletion, and the greater expression of AT_2_R has been seen in SHR than age-matched Wistar-Kyoto rats [9, 74, 75]. There are two AT_1_R subtypes, AT_1A_R and AT_1B_R. Both of them and AT_2_R mRNA are located in the afferent arteriole, arcuate artery, and outer medullary descending vasa recta [9, 76]. The expressions of AT_1A_R and AT_1B_R mRNA are similar in the glomerulus, while the glomerulus is the only structure with a relatively high AT_1B_R mRNA content, and the contribution of AT_1A_R is greater in all nephron segments [76, 77]. The MasR is also detected in both cortical and medullary regions of the kidney, the tubular and glomerular cells and vascular endothelium, afferent arterioles, proximal and distal tubules, collecting ducts, and thick ascending limb of Henle [78–81].

Hypertension and enhancement in Ang II levels may affect the distribution and regulation of intrarenal Ang II receptors, which cause different tubular and vascular receptor responses [69, 82] (Table 1). In hypertension, the AT_1_R and AT_2_R expressions are also tissue- and gender-dependent, and they are altered in SHR diabetic rats [75, 83–85]. It leads to higher mRNA and protein expressions of AT_1_R in the aorta of hypertensive rats compared to normotensive ones, while the AT_2_R expression remained unchanged (Table 1), and in diabetic and hypertensive rats, the mRNA and protein expressions of AT_1_R and AT_2_R are increased [86]. It is also reported that two weeks of Ang II infusion do not alter total kidney AT_1_R mRNA levels and receptor proteins [87]. However, within 7-day 2K1C and 3-day 2K1W rats, the AT_1_A receptor reduces in clipped or wrapped kidneys and contralateral kidneys when compared with normal animals [88]. Also, a 10-day systemic Ang II-induced hypertension decreases AT_1A_R protein in clipped and unclipped kidneys of 2K1C and in two kidneys, one wrap hypertensive model [82]. After two weeks of clipping (2K1C hypertensive rats), the glomerular AT_1_R decreased but vascular AT_1_R was not decreased until 16 weeks [88] (Table 1). Elevation of circulating and kidney Ang II levels in severe hypertension in Ren-2 gene transgenic rats increases AT_1_R binding in the vascular smooth muscle of afferent and efferent arterioles, juxtaglomerular apparatus, glomerular mesangial cells, proximal tubular cells, and renomedullary interstitial cells [89]. In 4-day 2K1C hypertensive Sprague-Dawley rats, the AT_1_R and AT_2_R expressions increase in the clipped kidneys without change in unclipped kidneys [90].

One week after performing the 2K1C model, the AT_1_R mRNA level decreases in the clipped kidney, but hypertension intensifies 10 weeks later and upregulation of AT_1_R mRNA levels in the clipped and unclipped kidneys occurs (Table 1). Upregulation of renal AT_1_R in multiple renal cells leads to Ang II hypersensitivity, chronic hypertension in the renovascular system, and the pathogenesis of hypertension [91, 92]. Also, in Ang II-dependent hypertension, vascular and glomerular AT_1_R is downregulated, but the proximal tubular receptors are either upregulated or not significantly altered [93] (Table 1).

AT_2_R is expressed in the adult kidney primarily in the renal proximal tubule cells [9, 74, 94] and inhibits renal Na^+^ reabsorption by internalizing and inactivating the major Na^+^ transporters (Na^+^-H^+^ exchanger-3 (NHE-3) and Na^+^/K^+^ ATPase) [94, 95]. 10-day systemic Ang II administration does not change the AT2R expression, but in 2K1C and 2K1W hypertensive models, AT2R is downregulated only in clipped kidneys but not in contralateral kidneys [82] (Table 1).

The MasR protein expression increases in the carotid of 2K1C rats [96]. The renal Mas mRNA is not different between SHR and normotensive Wistar rats (Table 1), while a 14-day infusion of Ang 1–7 decreases renal Mas mRNA expression in the SHR model [45]. According to a study, 2K1C reduced MasR in proximal tubules of the clipped kidney while AT1R was not suppressed; thus, the elevation in AT1R/MasR ratio was observed [97]. Although MasR was suppressed in the male sex [97], enhancement in MasR expression in both kidneys was seen in female rats 5 weeks after 2K1C [98]. As noted above, hypertension affects the distribution of Ang II receptors in the vessels and the kidneys. In the first days after Ang II-dependent hypertension induction, AT1R distribution decreases and may accompany the water and Na^+^ retention [24]. Also, it seems that in 2K1C and 2K1W hypertensive models, the distribution of the receptors in contralateral kidneys is similar to the kidneys in Ang II-dependent hypertensive subjects, so we have seen that the alterations of AT_1_R and AT_2_R in Ang II-infused rats are akin to contralateral kidneys. Also, it should be noted that the decrease in AT_1_R without altering AT_2_R in the kidney increases the AT_2_R/AT_1_R ratio. The decreased AT1R/MasR ratio has also been observed in male rats, while in the female sex, the expression of MasR increased, which results in different kidney responses to various factors in a patient with angiotensin-induced hypertension.

## 7. Inflammation in Ang II-Induced Hypertension

**Figure 2:**
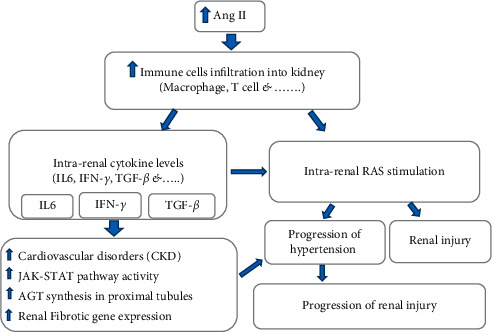
Ang II amplifies renal injury via inducing infiltration of immune cells into kidneys and enhancing intrarenal cytokine levels. On the other hand, the accumulation of immune cells in the kidneys causes intrarenal RAS stimulation which begins a cycle of injury again.

Ang II amplifies renal injury via lymphocyte response stimulation [99]. Elevated Ang II induces infiltration of immune cells into kidneys, leading to an increase in the intrarenal cytokine levels [44]. The accumulation of immune cells in the kidneys causes intrarenal RAS stimulation, progression of hypertension, and renal injury [100]. The proinflammatory effects of Ang II can also involve T cells, and chronic administration of Ang II infusion enhances macrophage and T-cell infiltration in the kidneys [101] (Figure 2). In the 2K1C model, macrophage and monocyte infiltration in unclipped kidney's glomeruli is greater than clipped or normal kidney, while the cortical interstitial macrophage infiltration in the cortex of both clipped and unclipped kidneys is greater than the normal one [23].

Activated immune cells produce several types of proinflammatory cytokines. The enhancement of RAS activity and the production of proinflammatory cytokines are synergized to induce hypertension [100]. Macrophages and T-cell infiltration into the kidney by Ang II-induced hypertension increase IL-6 [102], and AGT synthesis in proximal tubules is stimulated via cytokines, IL-6, and interferon-gamma (IFN-*γ*) [44, 56] (Figure 1). IL-6 is a multifunctional proinflammatory cytokine that intensifies CKD [103]. IL-6 activates Janus kinase/signal transducers and activators of transcription (JAK-STAT) pathway synergistically with Ang II, and it can motivate AGT creation, and the active AGT may be a crucial mechanism underlying elevated intrarenal AGT levels during Ang II-induced hypertension [44, 56, 57] (Figure 2). Ang II also causes IL-6 induction in the mouse kidney and genetic deletion of IL-6 significantly reduced blood pressure and renal injury and progression of renal fibrosis in angiotensin II-infused animals [100, 104]. IL-6 is also a key cytokine in downstream signaling of Ang II that induces fibrosis gene expression in the kidney and accelerates the progression of hypertension and consequent renal damage [102, 104]. In the Goldblatt hypertensive rat model, the level of IL-6 in the unclipped kidney is greater than in clipped kidneys and sham-operated kidneys [105].

Ang II stimulates intrarenal production of the profibrotic molecule transforming growth factor-beta (TGF-*β*) which develops kidney fibrosis [101, 106]. It is directly stimulated by the activation of AT_1_R [106]. In addition, the stimulation of lymphocytes may also be effective in the regulation of TGF-*β* in kidney disease [99] (Figures 1 and 2).

IFN-*γ* is one of the most important known proinflammatory factors. During Ang II-induced hypertension, the RAS stimulation enhances IFN-*γ* formation in activated T cells [99, 107], and it increases AGT expression in proximal tubules [108] (Figures 1 and 2). In contrast, TNF-*α* suppresses AGT expression in human proximal tubule cells while it enhances AGT level in several tissues such as the liver, aorta, and adrenal [109, 110]. The TNF-*α* infusion also increases urine volume (UV) and sodium excretion rates (UNaV), and it suppresses blood pressure and possibly the RAS activity [111].

In the proximal tubules, the AGT synthesis is stimulated by Ang II-induced proinflammatory factors, particularly IL-6 and IFN-*γ* derived from immune cells, which may include primary mechanisms underlying elevated intrarenal AGT levels during Ang II-induced hypertension.

## 8. Kidney Injury Markers in Ang II-Induced Hypertension and the Role of Fibrotic and Inflammatory Factors

It is well documented that the clipped kidney's weight is lower than unclipped and normal ones [23, 49], and the dry weight of unclipped kidneys is greater than normal in the 2K1C model [112]. In the unclipped kidney, the glomerular expansion and cell proliferation are greater than clipped or normal kidneys while in clipped kidneys it is slightly greater than the normal one [23]. Both clipped and unclipped kidneys obtain greater medullary fibrosis compared with normal kidneys [23]. The thickness of the vascular wall in the cortex of both clipped and unclipped kidneys is greater than the normal one, and the tubular epithelial cells of unclipped kidneys indicate signs of mesenchymal transition [23]. There is a mesangial expansion and renal fibrosis in the 2K1C rat model [49]. The unclipped kidney has a higher mesangial expansion, while the clipped kidney showed greater glomerular fibrosis [49]. The higher fibrosis found around the juxtamedullary resistance vessels and juxtamedullary cortex in the unclipped kidney of the 2K1C rat model indicates the role of pressure-induced injury to the vascular system or causes renovascular injury, and there are also greater cell proliferation, macrophage infiltration, fibrosis, glomerular expansion, and mesenchymal transition of tubular epithelial cells in the unclipped kidney [102, 113].

The inflammatory cells existed in the kidneys of RAS-induced hypertension and CKD [4, 114, 115]. The infiltration of immune cells into the kidney aggravates hypertension and renal damage while the activation of immune system inhibiting can improve blood pressure and renal disease [4, 116, 117]. During the progression of kidney fibrosis and hypertension, the macrophages and T lymphocytes accumulate around the renal vasculature and throughout the kidney's interstitium and provide an environment of proinflammatory and prohypertensive molecules that intensify tissue damage [4, 118, 119].

Exposure to the higher AGT induces a higher intratubular Ang II, a higher arterial pressure, and an enhancement of inflammatory cell infiltration and performs severe renal injury in the unclipped kidney, including glomerular expansion, medullary fibrosis, immune cell infiltration, and cell proliferation.

## 9. Renal Interstitial Fluid BK, NO, and cGMP Responses in Ang II-Induced Hypertension

In normal conditions, the renal interstitial fluid (RIF), BK, NO, and cGMP response to AT_1_R, AT_2_R, or BK B_2_-receptor antagonists administration do not alter; however, these antagonists alter the levels of the RIF BK, NO, and cGMP in wrapped and contralateral kidneys differently [14]. Losartan as AT_1_R antagonist enhances RIF BK, NO, and cGMP in the contralateral kidney, but not in the wrapped kidney. The infusion of PD123319 (AT2R antagonist) or coadministration of losartan and PD123319 reduces RIF BK, NO, and cGMP in both wrapped and contralateral kidneys [14]. In contrast, BK B_2_-receptor antagonist or coadministration of losartan and BK B_2_-receptor antagonist increases RIF BK in wrapped and contralateral kidneys, and BK B2-receptor antagonist decreases NO and cGMP in the both wrapped and contralateral kidneys [14]. The 4-day clipped kidneys in the 2K1C model show significant decreases in renal NO and cGMP levels and compound 21 (C21, AT2R agonist) causes a significant increase in NO and cGMP levels [90].

Ang II-induced renal vasoconstriction stimulates the release of a variety of vasoactive compounds from endothelial cells. The induced shear stress by the enhancement of renal perfusion pressure (RPP) causes NO production, which acts against autoregulation of RBF [120]. It is reported that sNG-nitro-L-arginine (L-NAME) infusion decreases RBF and increases mean arterial pressure (MAP) and RVR in normotensive and 2K1C hypertensive rats [121].

In the unclipped kidney of 2K1C hypertensive rats, such plateau part of the autoregulation curve is not observed compared to normotensive rats [121]. L-NAME infusion in the 2K1C model performs a higher degree of compensation in the unclipped kidney. The lower limit of RBF autoregulation is higher in 2K1C rats, and L-NAME infusion reduces it [121]. So, we can conclude that the regulation of RBF in the unclipped kidney is NO-dependent. In addition, NO inhibition enhances the efficacy of autoregulation of RBF in unclipped kidneys of 2K1C hypertensive rats' model while NOS inhibition in normotensive rats' model fails the efficacy of autoregulation [122, 123].

## 10. Renal Function and Hemodynamic Response in Ang II-Induced Hypertension

The CKD has been characterized based on the level of glomerular filtration rate (GFR), RBF, renal plasma flow (RPF), urinary potassium excretion rates (UKV), UV, UNaV, and the presence or absence of evidence of renal injury [5, 23, 112].

Surprisingly in one study on the 2K1C model, no differences in RBF, RPF, GFR, and UKV between clipped and unclipped kidneys with normal kidneys are detected [23], but the reduction of RBF and enhancement of RVR in 2K1C hypertensive rats are reported by others [121]. The renal cortical blood flow (RCBF) and renal medullary blood flow (RMBF) are not different between unclipped and normal kidneys, but RVR in the unclipped kidney is slightly increased [112]. The UV and UNaV in the unclipped kidney are higher than those in the normal kidney [23]. The decrease of UV in the wrapped kidney and the decrease of UNaV in both kidneys are observed in Grollman hypertensive rat models [14]. Losartan administration increases UV and UNaV in wrapped and unwrapped kidneys, and PD123319 and BK B_2_-receptor antagonist decrease UV and UNaV in the unwrapped kidney, while coadministration of losartan and PD123319, losartan, and BK B_2_-receptor antagonist abolishes the effect of losartan [14].

Also, RBF and RCBF are higher in the contralateral kidney compared to the wrapped one in the Grollman hypertensive model [14]. RBF does not change in control animals in response to AT_1_R, AT_2_R, or BK B2-receptor antagonist whereas RBF and RCBF increase in response to losartan in both wrapped and contralateral kidneys, while PD123319 and BK B2-receptor antagonist decrease RBF and RCBF in contralateral kidneys [14]. Coadministration of losartan and PD123319 decreased RCBF in the wrapped kidneys, but in the contralateral kidneys, PD123319 abolishes the effect of losartan and increased RCBF. BK B2-receptor antagonist, either alone or combined with PD123319, decreased RCBF and blocked the vasodilatory effect of losartan in the contralateral kidney [14].

The renal arterial Ang II infusion in normal conditions causes dose-dependent decreases in RCBF but not in RMBF, and PD123319 administration indicates a dose-dependent decrease in RBF and increases in RMBF response to Ang II, while candesartan (AT_1_R antagonist) eliminated all effects of Ang II (112). Changes in renal hemodynamics in response to renal arterial Ang II infusion were greatly diminished in 2K1C rats in comparison with normal conditions; for example, RMBF did not have any change in response to Ang II in presence of PD123319 [112]. Thus, AT_1_R mediates medullary vasodilatation, and in 2K_1_C hypertension, AT_2_R activation has a vasoconstrictive effect in medullary circulation [112].

The effects of acute AT_1_R blockade are dependent on AT_2_R activation. Candesartan reduces MAP and induces renal and mesenteric vasodilation, and coadministration of PD123319 and candesartan moderately reverses the depressor effects of candesartan in normotensive male rats but not SHR or 2K1C rats [112]. Moreover, in male and female SHRs, the AT_2_R stimulation increases RBF in females but not in males; however, it reduces RVR in females, without influencing MAP [124] whereas stimulation of AT_2_R in normotensive rats enhanced RBF in both males and females [125]. Also, UNaV enhancement in the absence of any major change in GFR is seen in female but not male hypertensive rats [124]. Renal AT_2_R expression in female rates is higher than that in male hypertensive rats [124], so, acute AT_2_R stimulation improves renal vasodilatation and sodium excretion without alterations in GFR in female hypertensive rats [124]. In addition, renal AT_1A_R expression is greater in female SHRs while renal AT_1B_R expression is not different between male and female SHRs [124].

## 11. Conclusion

The major findings of this review may be summarized as follows. First, the local RAS receptor distributions and AGT expressions may be altered in the unclipped kidney. Second, the increase of the local level of ACE and the decrease of ACE2 activities occur which lead to the enhancement of Ang II and reduction of Ang 1–7 in Ang II-dependent hypertension. Third, the alteration of local cytokines level followed by an enhancement in immune cell infiltration promotes kidney dysfunction and injury in Ang II-dependent hypertension with the greater renal injury in the unclipped kidney. Fourth, the renal function of the unclipped kidney is associated with alteration of NO, BK, and cGMP that reveals the important role of kidney endothelial function during hypertension. Finally, the renal functions and hemodynamic responses to Ang II-induced hypertension may differ from normal conditions which imply the association between induced hypertension and hemodynamic alteration that affected renal functions.

In the 2K1C model, factors that affect the unclipped kidney are more similar to the kidneys of the Ang II-infused models. Therefore, unclipped kidneys in 2K1C or unwrapped kidneys in 2K1W models and similarly the Ang II-infused models are more suitable examples to refer to people with Ang-dependent hypertension. It should be noted that in the 2K1C model although systemic effects are similar in both kidneys at risk, the clipped kidney is protected against the induced hypertension, and the unclipped kidney is subjected to higher RPP, and the elevated RPP has a dominant role in renal injury or barotrauma [23, 113].

In addition, the unclipped kidneys in the 2K1C model indicated the greater glomerular expansion and immune cell infiltration, medullary fibrosis, and cellular proliferation which is possibly related to the synergic role of pressure-induced injury and intrarenal Ang II-exacerbated renal injury [23, 102, 113].

On the other hand, following an increase in the immune cell infiltration, the level of cytokines increased in the unclipped kidney which also is accompanied by renal injury [105].

The uAGT excretion rate in hypertensive patients is greater than in normal subjects [44, 52]. As mentioned before, this phenomenon is similar to the unclipped kidney in the 2K1C rat model where the intrarenal AGT mRNA level, uAGT excretion rates, and uAGT/urinary protein excretion ratio in the unclipped kidney were greater than those in the clipped kidney [23, 53]. AGT overexpression in the proximal tubule increased the generation of ROS, NADPH oxidase activity, tubular apoptosis, and tubulointerstitial fibrosis, which affect renal function and degree of injury [6, 23, 67]. Elevated renal interstitial Ang II concentration due to AGT secretion also contributes to sustaining stimulation of sodium reabsorption, vasoconstriction, development of hypertension, and progressive renal injury and fibrosis [22, 23, 44]. Collectively, it seems that local AGT and Ang II increasing, Ang 1–7 decreasing, and those factors involved in the accumulation of immune cells into kidneys, leading to an increase in the intrarenal cytokine levels, are the main factors that promote the degree of injury in the unclipped kidney; therefore, the control of these parameters in hypertensive patients with a high level of Ang II is extremely important.

The RAS receptor functions are also involved in hypertension development. In contralateral kidneys of 2K1C and 2K1W and the kidneys of Ang II-infused hypertensive models, the reduction in AT1R without change in AT2R and therefore the enhancement in AT2R/AT1R ratio were detected [82, 88]. The MasR receptor was also affected by hypertension sex dependently [97, 98]. As mentioned before, in the 2K1W hypertensive model, the local RIF BK, NO, and cGMP in the contralateral kidney were higher than normotensive rats [14] which are affected by AT2R and AT1R activity, because losartan increases and PD123319 decreases RIF BK, NO, and cGMP in the unwrapped kidney of 2K1W hypertensive rats, while such observations were not seen in normotensive rats when losartan and PD were administrated [14]. To support this idea, it is reported that AT2R stimulation leads to an increase in the levels of BK, NO, and cGMP [126], where the last one increases via BK2 receptor and activates NO, and PD123319 administration diminishes RBF and GFR [126, 127]. Therefore, it seems that the receptor function absolutely depended on the condition of hypertension and normotension, and the interaction between receptors may develop different functions in the unclipped kidney in the 2K1C model or hypertensive subject with high Ang II.

Ultimate alteration in Ang II to Ang 1–7 ratio, change in AT2R/AT1R ratio, MasR expression, shear stress, inflammatory factors, and the elements which are affected by these factors such as NO, BK, and cGMP affected the basic autoregulation and RBF and alter renal response in different situations in Ang II-dependent hypertension.

## Figures and Tables

**Table 1:** RAS receptor distribution change in response to hypertension. AT_1_R distribution change related to time after 2K1C or 2K1W in hypertensive rat models.

Major RAS receptors		Hypertension	
AT_1_ receptor		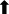 Aorta AT_1_R expression Vascular and glomerular AT_1_R downregulation Proximal tubular receptors upregulation or not significantly altered	
AT_1_ receptor alteration after 2K1C or 2K1W in hypertensive rat models		*Time after clipping*	
	1 week	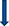 AT_1_R mRNA in the clipped, wrapped, and contralateral kidneys
	10 days	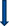 AT_1A_R protein in clipped and unclipped kidneys
	2 weeks	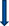 Glomerular AT_1_R
	10 weeks	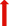 AT_1_R mRNA levels in clipped and unclipped kidneys
	16 weeks	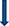 Vascular AT1R
AT_2_ receptor		Aorta AT_2_R expression 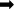 unchanged Sodium depletion & SHR 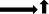 AT_2_R expression AT_2_R downregulation only in clipped and wrapped kidneys, in 2K1C and 2K1W	
Mas receptor		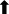 Carotid MasR protein expression in 2K1C rats 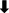 MasR in proximal tubules of clipped kidney in male rats 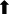 MasR in clipped and unclipped kidney of female rats	
